# Multi-characterization-assisted construction of the molecular structure of high-volatile bituminous coal

**DOI:** 10.1371/journal.pone.0354266

**Published:** 2026-07-20

**Authors:** Xiang Feng, Youquan Dou, Yuanyuan An, Zheyao Xiong, Qingsong Wang, Tan Shi, Rudan Chen

**Affiliations:** 1 National Environmental Protection Research Institute for Electric Power Company, Limited, Nanjing, Jiangsu, China; 2 Guoneng Nanjing Coal Quality Supervision and Inspection Company, Limited, Nanjing, Jiangsu, China; 3 Hangzhou Hikvision Digital Technology Company, Limited, Hangzhou, Zhejiang, China; 4 Zhejiang University, Hangzhou, Zhejiang, China; University of Sahiwal, PAKISTAN

## Abstract

Elucidating coal molecular structures is critical for studying its structure–property relationships and advancing efficient coal resource utilization. In this study, a bituminous coal (JSM_C) was selected from the coal-rich Inner Mongolia region of China as the research object. Multi-characterization technologies, including elemental analysis, ^13^C solid-state nuclear magnetic resonance spectroscopy (^13^C NMR), Fourier transform infrared spectroscopy (FTIR), and X-ray photoelectron spectroscopy (XPS), were integrated to characterize the elemental composition, carbon skeleton, functional groups, and nitrogen species in JSM_C. The results showed that the molecular formula of JSM_C is C_176_H_128_O_19_N_2_. The carbon skeleton is centered on mono-/bi-/tricyclic aromatics connected by aliphatic or oxygen-linked chains. The oxygen-containing functional groups are mainly phenols and ethers, while nitrogen species exist in the form of pyridine and pyrrole heterocycles. The simulated spectra of NMR and FTIR are consistent with the experimental data, confirming the reliability of the constructed structure. This study achieved the construction of complex coal macromolecules from the specific mine through “characterization analysis-model construction-simulation verification,” providing a feasible paradigm for coal molecular structure research. It also lays a molecular foundation for investigating structure-performance relationships and introducing related algorithmic models in coal research.

## 1. Introduction

Coal, as an important primary energy source and chemical feedstock, plays a critical role in achieving energy-structure transformation and carbon-neutrality goals [1,2]. Coal utilization performance is mainly determined by its skeletal structure and functional groups [3]. Although coals of the same rank generally possess similar macroscopic characteristics, significant microstructure heterogeneity still exists among coals from different production areas due to the influences of geological evolution histories, coal-forming pathways, and depositional environments ^[^4–8^]^. This difference will be further amplified during conversion processes such as pyrolysis, gasification, and liquefaction, thereby affecting their application performance. For instance, Zhang et al.[8] reported that coking coal from Xinjiang contains more aliphatic side chains and highly reactive oxygen-containing functional groups in its macromolecular structure than North China coal of the same rank. Consequently, the resulting coke exhibits a lower coke strength after reaction (CSR), failing to meet the operational requirements of large and medium blast furnaces (above 1000 m³). Therefore, investigating the molecular structure of coal from specific mining areas holds great significance for the customized development and efficient utilization of coal resources.

Methodologies for investigating coal molecules have continually evolved, with traditional studies predominantly relying upon chemical analysis and basic spectroscopic characterization. Common characterizations, such as ultimate analysis, nuclear magnetic resonance (NMR), and Fourier-transform infrared spectroscopy (FTIR), are widely utilized to reveal the elemental composition, skeletal frameworks, and functional groups of coal molecules [4,9]. Subsequently, the rapid advancement of computational chemistry and molecular simulation technologies, coupled with traditional methods, has further enhanced the accuracy of constructing molecular models [1,10]. In recent years, the rapid iteration of artificial intelligence technologies has demonstrated substantial potential for applications within the coal industry [11,12]. For example, spectroscopic techniques combined with machine learning-based recognition models can enable rapid and accurate compositional analysis of different coal types ^[^13–15^]^. However, for deep chemical reaction processes such as pyrolysis, which involve complex bond cleavage, existing end-to-end black-box models often fail to deliver satisfactory predictive performance [16]. The fundamental reason is that the coal pyrolysis behavior is governed by its highly complex macromolecular structure. Therefore, models must rely on sufficient structural data to establish an intrinsic structure–property relationship to improve both prediction accuracy and generalizability. Despite over 130 published average molecular models for coal, structural data for specific mining areas, such as the Balongtu mine area, remain scarce, which restricts the accurate assessment of coal applications and performance prediction in this region [15,17–19^]^.

Based on the aforementioned, this study focuses on coal samples collected from the Balongtu mine area in Inner Mongolia, China. Situated within the “Energy Golden Triangle” region, this area boasts abundant coal resources, yet systematic investigations at the molecular level remain conspicuously absent. The coal sample was denoted as JSM_C, and systematically analyzed via multi-characterization techniques, including ^13^C NMR, FTIR, elemental analysis, and XPS. Subsequently, a three-dimensional (3D) structure of the coal molecule was constructed based on specific construction rules, and its energy was optimized using the molecular mechanics method. Finally, the ^13^C NMR and FTIR spectra of the 3D molecule were simulated to refine and validate the model against the experimental data. This spectral simulation ensured that the constructed 3D molecular structure was consistent with the measured constraints, establishing it as one of the representative average structural models for this coal type and laying a solid foundation for subsequent property investigations.

## 2. Materials and Methods

### 2.1. Materials preparation

The coal samples used in this study were commercial coal, purchased from China Energy Trading Group Co., Ltd., and were assembled and dispatched from the Batuta Station. Therefore, no specific field site access or environmental permits were required for this work. The coal was originally collected from the Balongtu coal mine in Nalintaohai Town, Eerduosi (Ordos) City, Inner Mongolia Autonomous Region, China. The mine is located within the Shanxi–Shaanxi–Inner Mongolia energy triangle region. The coal sample was crushed, mixed, and divided in accordance with the Chinese National Standard GB/T 474‑2008 *Method for preparation of coal sample*, yielding coal particles with a nominal size of 3 mm. Subsamples were randomly selected multiple times for subsequent experimental use.

### 2.2. Proximate analysis and elemental analysis

Proximate analysis of the coal samples was carried out according to the Chinese National Standard GB/T 212–2008 *Proximate analysis of coal*, to determine the contents of moisture, ash, volatile matter, and fixed carbon. Elemental analysis was performed following the relevant Chinese National Standards: GB/T 476–2008 *Determination of carbon and hydrogen in coal*, GB/T 19227–2008 *Determination of nitrogen in coal*, and GB/T 214–2007 *Determination of total sulfur in coal*.

### 2.3. Fourier transform infrared spectroscopy (FTIR) test

The coal sample was thoroughly ground and sieved through a 400-mesh standard sieve. The sieved sample was uniformly mixed with KBr and pressed into thin pellets (thickness: ~ 0.2 mm) for FTIR testing. The spectral measurement was conducted over a wavenumber range of 400–4000 cm ⁻ ¹ with 48 scans.

### 2.4. X‑ray photoelectron spectroscopy (XPS) test

The coal sample was thoroughly ground and sieved through a 400-mesh standard sieve. X-ray photoelectron spectroscopy (XPS) measurements were performed on an ESCALAB 250Xi spectrometer (Thermo Scientific) using a monochromatic Al-Kα X-ray source (1486.7 eV). The powder sample was fixed on double-sided tape and pressed into pellets. We first acquired a full survey scan (0–1400 eV), then performed high-resolution narrow scans for C, O and N. The Lens mode was operated in the standard mode.

### 2.5. Carbon-13 nuclear magnetic resonance (13C NMR) test

The coal sample was thoroughly ground and sieved through a 400-mesh standard sieve. Solid-state ¹³C nuclear magnetic resonance (^13^C NMR) spectra were acquired on a Bruker Avance Neo 600 MHz spectrometer equipped with a 4 mm magic angle spinning probe (PhoenixNMR NB HXY). The experiments were performed using the cross-polarization (CP) with total sideband suppression (TOSS) technique. The spectral width was set to 58.8 kHz with a relaxation delay of 5.0 s, 4000 scans, and an observation frequency of 150.93 MHz.

### 2.6. Calculation method

The molecular structure was constructed using KingDraw software. Structural optimization was performed with the Forcite module (Dreiding force field) in Materials Studio, with the maximum iteration steps set to 60000 and energy convergence tolerance of 0.001 kcal/mol. The Fourier transform infrared (FTIR) vibrational spectrum was then simulated utilizing the DMol3 module. Additionally, the NMR spectra were simulated by the built-in function of Mnova.

## 3. Results and discussion

### 3.1. Confirmation of molecular formula

Proximate analysis reveals that the air-dry basis ash content of JSM_C is 7 wt%. Its dry ash-free volatile matter and carbon content reach 37 wt% and 82 wt%, respectively. In accordance with Chinese National Standard GB/T 5751–2009 *Chinese Classification of Coals*, JSM_C is identified as high-quality bituminous coal characterized by low coalification degree and ultra-low ash yield ^[^20–22^]^. Generally, sulfur, which has the lowest content and a high relative atomic mass, is chosen as the reference element to deduce the molecular formula. For JSM_C, the sulfur content is only 0.3 wt% (Table 1). Such a low sulfur content would result in an excessively large number of atoms in the deduced molecule, which would not only greatly increase the difficulty of structural construction but also contradict the information of a relatively low degree of coalification. Therefore, nitrogen was selected as the reference element, consistent with reports in the literature [23,24]. When the number of nitrogen atoms in a molecule is 1, 2, or 3, the corresponding molecular weights are 3889, 2593, and 1296, respectively. Li et al.[25] pointed out that the molecular weight of coal is approximately 2000–3000. In the study of Jing et al.[20], the molecular weight of high-volatile bituminous coal is about 2500. Hence, the number of nitrogen atoms was determined to be 2. Since the sulfur content is extremely low and the number of sulfur atoms is less than one in a single molecular structure, sulfur elements are temporarily excluded in the structural construction. Therefore, the molecular formula of JSM_C was confirmed to be C_176_H_128_O_19_N_2_, and the molecular weight was 2572 (without sulfur).

**Table 1:** Ultimate analysis of JSM_C.

Ultimate analysis (wt%, daf)^1^				
C	H	O	N	S
81.69	4.96	11.94	1.08	0.34

^1^Mass fractions of different elements obtained under the dry ash-free (daf) basis.

### 3.2. Analysis of carbon skeleton

**Fig 1:**
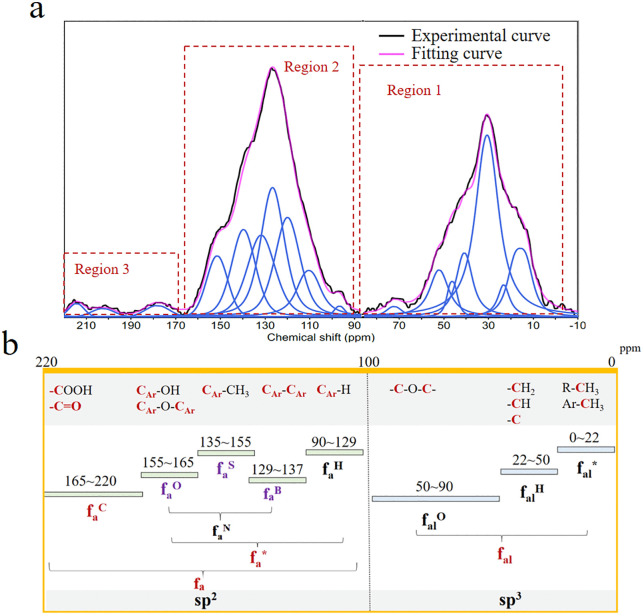
NMR spectrum analysis and structural parameter diagram. (a) Experimental and fitted curves of ^13^C NMR of JSM_C; (b) Schematic diagram of chemical shift ranges and structural parameters corresponding to carbon in different chemical environments.

Solid-state ¹³C NMR can directly identify the chemical environment of carbon atoms, so it is widely applied to characterize the carbon skeleton of coal macromolecules [20]. Fig 1a shows the ^13^C NMR spectrum of JSM_C, which is mainly split into three regions corresponding to aliphatic carbon peaks (0–90 ppm), aromatic carbon peaks (90–165 ppm), and carboxyl and carbonyl carbon peaks (165–220 ppm) [18,23,26,27]. The carbon types can also be classified based on SP^2^ and SP^3^ hybridization (Fig 1b) [28]. In the aliphatic carbon peak region, the chemical shifts from low to high correspond to methyl, methylene, methine, quaternary, and ether carbons, respectively. Of these, methine and quaternary carbons cannot be completely distinguished, while methylene carbon is dominant, accounting for 21.8% of total carbon atoms. The aromatic carbon region corresponds to protonated aromatic carbon, bridged carbon, alkylated aromatic carbon, and oxygen-bonded aromatic carbon. Among these, protonated aromatic carbon had the highest content, accounting for 30.6%. Carboxyl and carbonyl carbons accounted for relatively low proportions, at 1.0% and 1.9%, respectively. Detailed peak information is listed in S1 Table.

There are twelve structural parameters of the coal molecule derived from NMR spectroscopy (Fig 1b and Table 2). Eight parameters directly correspond to carbon structural units, namely f_al_^*^ (methyl carbon), f_al_^H^ (methylene/methine/quaternary carbon), f_al_^O^ (ether carbon), f_a_^H^ (protonated aromatic carbon), f_a_^B^ (aromatic bridgehead carbon), f_a_^S^ (alkylated aromatic carbon), f_a_^O^ (oxygen-bonded aromatic carbon), and f_a_^C^ (ketone/carboxyl carbon). Furthermore, the remaining four carbon parameters, derived from the aforementioned eight, are f_a_^N^ (non-protonated aromatic carbon), f_al_ (total sp^3^-hybridized carbon), f_a_^*^ (total aromatic carbon), and f_a_ (total sp^2^-hybridized carbon). In light of the molecular formula of JSM_C, the number of different carbon structure types in a single molecule was calculated (Table 2), which served as the basis for constructing the coal molecule.

**Table 2:** Structural parameter analysis of JSM_C from ^13^C NMR spectra.

Structural parameter	Content (%)^1^	Amount of carbon^2^
f_al_	43.2	77
f_al_^H^	28.9	51
f_al_^*^	8.7	16
f_al_^O^	5.6	10
f_a_	56.8	100
f_a_^C^	2.9	5
f_a_^*^	53.9	95
f_a_^H^	30.6	54
f_a_^N^	23.3	41
f_a_^O^	4.9	9
f_a_^S^	8.7	15
f_a_^B^	9.7	17

^1^The proportions of different structural parameters are calculated from the integration of NMR spectral peaks.

^2^The amount of different types of carbon in a single molecule is calculated based on the molecular formula (C_176_H_128_O_19_N_2_).

**Fig 2:**
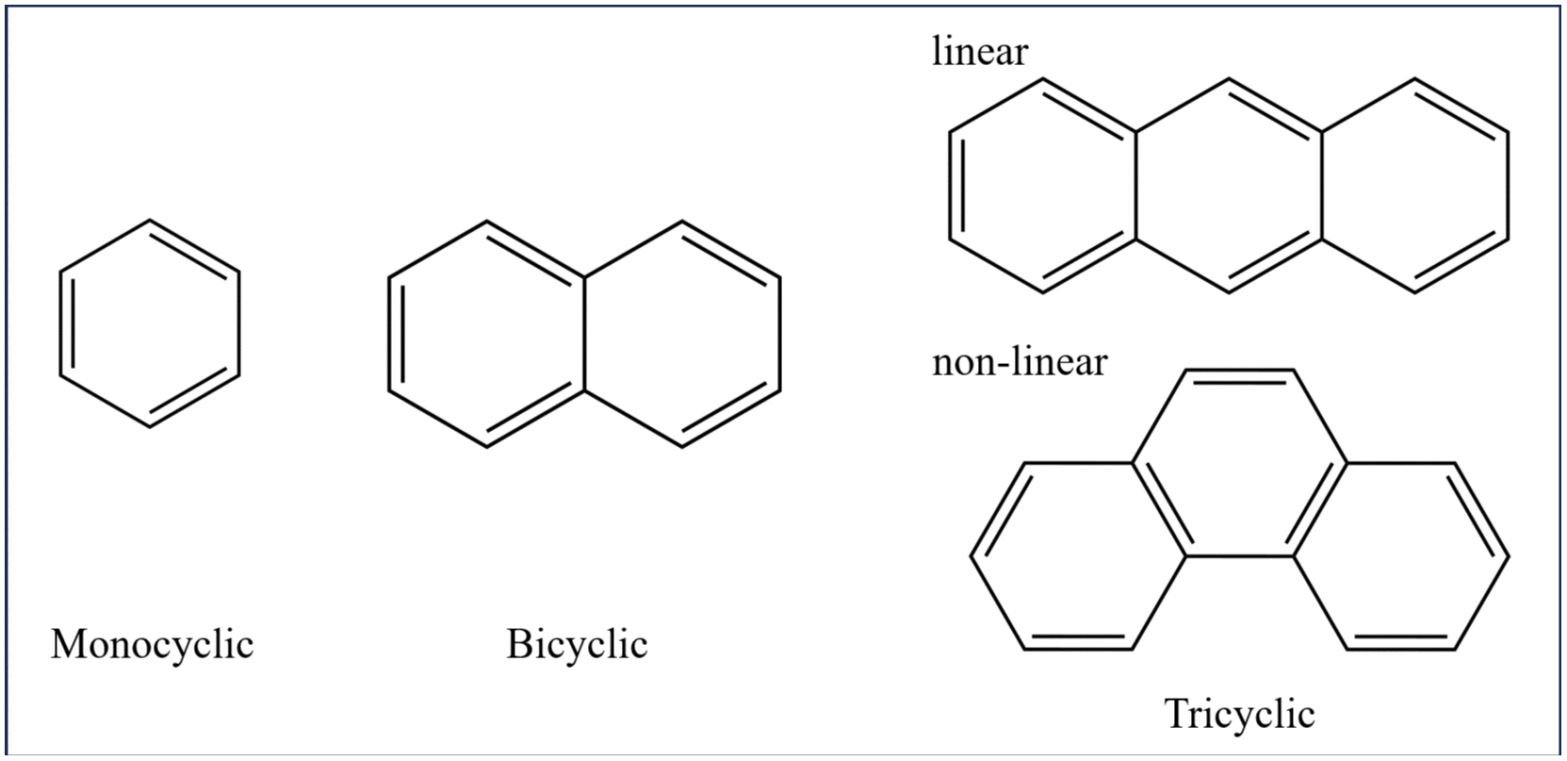
Types of aromatic rings.

Generally, the aromatic rings of coal molecules are composed of stable six-membered rings [29]. During coal evolution, higher coalification degree corresponds to more six-membered rings and a larger proportion of aromatic carbon [4,30]. Six-membered rings are capable of forming clusters of different sizes, which are connected through aliphatic or oxygen-linked chains to form the coal molecule. Solum et al.[31] pointed out that the size of clusters is related to the ratio (χ_b_) of bridgehead carbon (f_a_^B^) to aromatic carbon (f_a_^*^). The χ_b_ value of JSM_C is 0.18, corresponding to an average of ~10 carbon atoms per cluster, indicating an average bicyclic configuration. In consequence, mono-/bi-/tricyclic aromatics are mainly adopted during structure construction, which is consistent with the conclusion of judging cluster size by fixed carbon content [28]. According to the χ_b_ value and structural parameters, a quantitative allocation of carbon clusters was performed, yielding 4 monocyclic, 3 bicyclic, 2 linear tricyclic, and 1 non-linear tricyclic clusters, respectively (Fig 2). The ratio of bridgehead carbon to aromatic carbon of 10 cluster units was 0.19, which was essentially consistent with the χ_b_ value of JSM_C (0.18).

### 3.3. Analysis of functional groups

**Fig 3:**
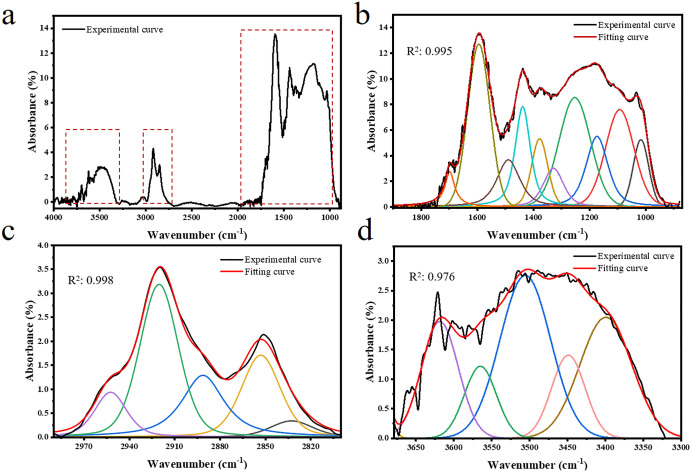
FTIR spectrum and fitting analysis. (a) FTIR spectrum of JSM_C; (b–d) Peak fitting results of FTIR spectra in (b) 1000–1800 cm ⁻ ¹, (c) 2800–3000 cm ⁻ ¹, and (d) 3300–3700 cm ⁻ ¹ regions. The black solid line represents the experimental curve, and the red one is the total fitted curve.

Fig 3 exhibits the FTIR spectra of JSM_C for characterizing the functional groups of the coal molecule. The FTIR spectrum (Fig 3a) is divided into three regions: 1000–1800 cm ⁻ ¹ (mainly oxygen-containing functional group vibrations), 2800–3000 cm ⁻ ¹ (mainly alkyl group vibrations), and 3300–3700 cm ⁻ ¹ (mainly hydroxyl group vibrations) [25,32–34^]^. Subsequently, peak fitting was performed rigorously (Figs 3b–d), and the fitting results are summarized in Table 3. The coefficients of determination (R²) for the peak fitting are 0.995 (1000–1800 cm ⁻ ¹), 0.998 (2800–3000 cm ⁻ ¹), and 0.976 (3300–3700 cm ⁻ ¹), respectively, indicating high-quality fitting results. The results reveal that oxygen-containing functional groups predominantly exist as phenols and aryl ethers, with alkyl ether content relatively low and acidic groups at the lowest level. For alkyl groups, methylene is the most predominant component, followed by methyl, while methine is the least abundant. And hydroxyl groups are mainly present as phenols and adsorbed free water. The analytical conclusions derived from these FTIR spectra are consistent with the analysis of NMR spectra.

**Table 3:** Peak fitting results of FTIR spectra of JSM_C.

Region (cm ⁻ ¹)	Functional groups	Center (cm ⁻ ¹)	Area (%)
1000-1800	R-O-R	1021	5.5
	Ar-O-Ar	1092	14.8
	Ar-OH	1175, 1253	37.7
	-CH_3_, -CH_2_	1373–1490	20.3
	C = C (aromatic)	1595	20.4
	-COOH	1698	1.3
2800-3000	-CH_3_	2853, 2950	32.6
	-CH_2_	2834, 2920	52.5
	-CH	2892	14.9
3300-3700	-OH···O (hydrogen bond)	3400, 3449	36.3
	Ar-OH	3505	32.9

### 3.4. Chemical environment of nitrogen

**Fig 4:**
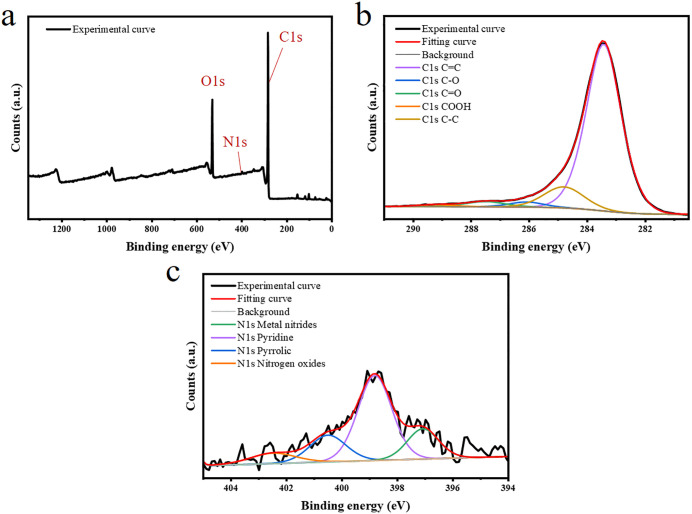
XPS spectra and fitting analysis. (a) XPS survey spectrum of JSM_C; (b–c) XPS spectra for (b) C 1s, and (c) N 1s. The black solid line represents the experimental curve, and the red one is the total fitted curve.

XPS technology was used to characterize the elemental chemical environments in coal. In the wide-scan XPS spectrum (Fig 4a), the major peaks are attributed to carbon and oxygen, indicating the high abundance of these two elements. The spectrum calibration was achieved by calibrating the binding energy of the C-C bond to 284.8 eV [32,35]. As shown in Fig 4b, the C 1s narrow-scan spectrum was obtained and then subjected to peak fitting, with binding energies from low to high corresponding to C = C (283.4 eV), C-C (284.8 eV), C-O (286.1 eV), C = O (287.5 eV), and COOH (289.2 eV). Peak signal intensities for carboxylic acid groups were extremely low, with their corresponding abundance also low, which was consistent with NMR and FTIR results. For the nitrogen spectrum, there are four chemical states assigned to metal nitrides (397.1 eV), pyridine (398.8 eV), pyrrole (400.5 eV), and nitrogen oxides (402.5 eV), respectively [36,37]. Notably, pyridine (six-membered heterocycle) and pyrrole (five-membered heterocycle) are the predominant nitrogen-containing structures in JSM_C, in line with well-established insights into coal molecular studies [4]. Nitrogen oxides, meanwhile, predominantly originate from small molecules, while metal nitrides mainly arise from the combination of nitrogen and residual transition metals.

In a word, XPS characterization reveals that nitrogen is predominantly present as pyridine (a six-membered heterocycle) and pyrrole (a five-membered heterocycle) in JSM_C.

### 3.5. Molecular structure construction and optimization

The construction and optimization of the coal molecule of JSM_C were performed as follows:

1. Linking ten cluster units of different sizes via 2–3 atom-long aliphatic chains or oxygen-linked chains [4,27].
2. Adding functional groups to the preliminary structure.
3. Introducing nitrogen moieties in the form of pyridine and pyrrole.
4. Simulating the NMR spectrum of the constructed molecule, comparing deviations between the simulated and experimental spectra, and then iteratively optimizing the molecular structure.

**Fig 5:**
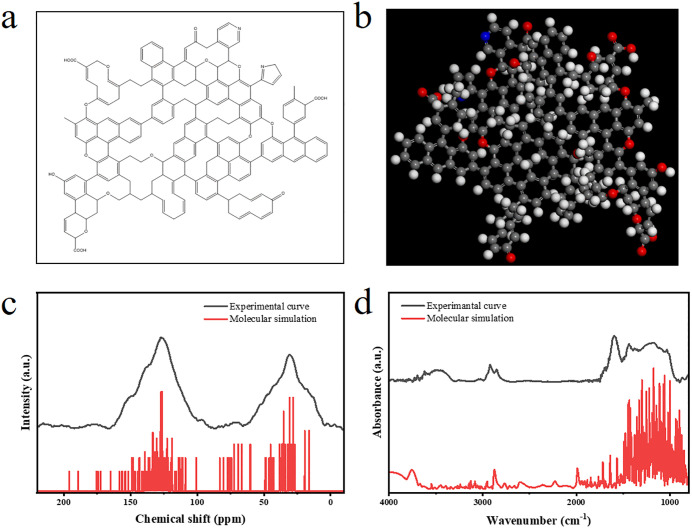
Schematic diagram of molecular structure and spectra simulation. (a) The optimized 2D molecular structure; (b) The 3D molecular structure after energy optimization; (c–d) Comparison of simulated and experimental (c) ^13^C NMR spectra, and (d) FTIR spectra.

As shown in Figs 5a and 5b, the 2D and 3D molecular structures are presented, which have been energy-optimized using molecular mechanics. It should be noted that coal is a highly heterogeneous and complex mixture, and its measured characterization data reflect average properties. Accordingly, the proposed molecular model is an average structure representative of the coal characteristics. The constructed molecule has a formula of C_176_H_128_O_19_N_2_ and a molecular weight of 2572, consistent with the previous elemental analysis (Table 4). Actually, the determination of this final molecular structure mainly relied on the NMR experimental data as a reference. Through multiple rounds of iterative optimization, the simulated NMR spectrum was ultimately consistent with the experimental spectrum (Fig 5c), thereby ensuring structural accuracy. A comparison of the key structural parameters (f_a_, f_a_^*^, f_al_) derived from simulated NMR spectra with experimental values is presented in Table 4. The absolute deviations of these parameters are less than 2%, which confirms that the optimized structure is consistent with the constraints from experimental measurements. However, to further verify the structure’s reliability, the FTIR spectrum of the constructed molecule was also simulated and compared against the experimental FTIR data. As the constructed molecule is an average molecular structure that primarily represents the characteristic information of the coal, the FTIR simulation verification focuses on the consistency of vibrational frequencies (peak positions). The results in Fig 5d demonstrate that the main peak positions of the simulated FTIR spectrum are fundamentally consistent with the experimental spectrum, confirming that the primary functional group structures are in agreement with the measured constraints.

**Table 4:** Comparison of experimental and simulation parameters.

	molecular formula	Structural parameters		
		f_a_ (%)	f_a_^*^ (%)	f_al_ (%)
JSM_C	C_176_H_128_O_19_N_2_	56.8	53.9	43.2
3D model	C_176_H_128_O_19_N_2_	56.9	54.8	43.1

In summary, analyses of the simulated NMR and FTIR spectra confirm that the constructed 3D molecular structure possesses structural features consistent with experimental constraints, confirming its reliability.

## 4. Conclusions

In this study, the coal molecular structure of the sample JSM_C was analyzed and constructed. The molecular formula was determined to be C_176_H_128_O_19_N_2_ with a molecular weight of 2572. By integrating characterization techniques such as NMR, FTIR, and XPS, the carbon skeleton, functional groups, and chemical structure of nitrogen were confirmed, and a molecular structure model was successfully constructed. Then, the constructed structure was optimized multiple times to ensure consistency between the simulated NMR spectrum and the experimental spectrum, thus guaranteeing the correctness of the molecular structure optimization. Finally, the FTIR spectrum was simulated, and the results were found to align with the experimental spectrum, further validating the reliability of the molecular structure model. In this work, a complete process from “multi-characterization structural analysis-model construction-spectral verification” was implemented, offering a viable framework for molecular structure research on analogous coal types and establishing a molecular basis for future performance investigations and the integration of algorithmic models in practical application.

## Supporting information

S1 Table Solid-state ¹³C NMR spectral analysis results of JSM_C. (DOCX)
