# Supplementary material for: Multi-characterization-assisted construction of the molecular structure of high-volatile bituminous coal
Source: PLoS One. 2026 Jul 20;21(7):e0354266. doi: 10.1371/journal.pone.0354266 (PMC13384289; doi:10.1371/journal.pone.0354266)
Supplement: S1 Table — (DOCX) [file pone.0354266.s001.docx]

**Supporting Information**

**Multi-characterization-assisted construction of the molecular structure of high-volatile bituminous coal**

Xiang Feng ^1,2^, Youquan Dou ^1,2^, Yuanyuan An ^3,4^, Zheyao Xiong ^3^, Qingsong Wang ^1,2^, Tan Shi ^1,2^, Rudan Chen ^3^*

^1^ National Environmental Protection Research Institute for Electric Power Company, Limited, Nanjing, Jiangsu, China

^2^ Guoneng Nanjing Coal Quality Supervision and Inspection Company, Limited, Nanjing, Jiangsu, China

^3^ Hangzhou Hikvision Digital Technology Company, Limited, Hangzhou, Zhejiang, China

^4^ Zhejiang University, Hangzhou, Zhejiang, China

*****Corresponding author

E-mail: [chenrudan@hikvision.com](mailto:chenrudan@hikvision.com) (RC)

| Peak number | Chemical shift (ppm) | Carbon species | Proportion (%) | Structural parameter |
| --- | --- | --- | --- | --- |
| 1 | 15.8 | methyl carbon | 8.72 | f_al_^*^ |
| 2 | 23.2 |  |  |  |
| 3 | 30.6 | methylene carbon | 21.85 | f_al_^H^ |
| 4 | 40.8 | methine/quaternary carbon | 7.06 | f_al_^H^ |
| 5 | 46.2 |  |  |  |
| 6 | 52.1 | ether carbon | 5.61 | f_al_^O^ |
| 7 | 72.3 |  |  |  |
| 8 | 96.7 | protonated aromatic carbon | 30.56 | f_a_^H^ |
| 9 | 110.4 |  |  |  |
| 10 | 119.9 |  |  |  |
| 11 | 126.7 |  |  |  |
| 12 | 131.7 | bridged carbon | 9.71 | f_a_^B^ |
| 13 | 139.7 | alkylated aromatic carbon | 8.71 | f_a_^S^ |
| 14 | 151.4 | oxygen-bonded aromatic carbon | 4.88 | f_a_^O^ |
| 15 | 178.2 | carboxyl carbon | 0.99 | f_a_^c^ |
| 16 | 202.6 | carbonyl carbon | 1.91 | f_a_^c^ |
| 17 | 214.3 |  |  |  |

**Table S1.** Solid-state ¹³C NMR spectral analysis results of JSM_C.
